# Joseph Fuller

**Published:** 1912-12

**Authors:** 


### JOSEPH FULLER, F.R.C.S. Ed.

THE large assembly at the Church of St. Mary, Portbury, on December 10th, at the burial of the deceased, who had resided and practised at Long Ashton for thirty-four years, showed how much Dr. Fuller was appreciated by his friends and patients. Qualified in 1876 at Trinity College, Dublin, he spent his whole professional life at Long Ashton, and won the esteem of all classes in the district. He held many public offices, and was Vice-Chairman of the Parish Council. He was a keen volunteer, and lately held the rank of Surgeon-Major (V.D.), Wessex Division, R.E. Territorial Force.

Like most of his fellow-countrymen, he was fond of the horse, and won many prizes at the local Agricultural Society, of which he was Vice-President. He took great interest in public health questions, was a Fellow of the Royal Institute, and was Medical Officer of Health for the district, as well as District Medical Officer for Long Ashton and Barrow Gurney since the year 1879.

Both in his professional duty and in his public life he was a man of great capacity, who served his generation well, and left his parish much better than he found it. He will be long remembered and mourned by all with whom he was associated, who regret that at the comparatively early age of 58 his life's work should have terminated.

